# Association of the inflammation indicators with multiple system involved of immunoglobulin A vasculitis in children

**DOI:** 10.3389/fped.2026.1921404

**Published:** 2026-09-14

**Authors:** Hao Li, Xinyi Shao, Huan Yang, Qiu Li

**Affiliations:** 1 Department of Dermatology, Children’s Hospital of Chongqing Medical University, National Clinical Research Center for Children and Adolescents’ Health and Diseases, Ministry of Education Key Laboratory of Child Development and Disorders, Chongqing, China; 2 Chongqing Key Laboratory of Child Rare Diseases in Infection and Immunity, Chongqing, China; 3 Department of Dermatology, The First Affiliated Hospital of Chongqing Medical University, Chongqing, China; 4 Department of Nephrology, Children’s Hospital of Chongqing Medical University, National Clinical Research Center for Child Health and Disorders, Ministry of Education Key Laboratory of Child Development and Disorders, Chongqing, China; 5 Chongqing Key Laboratory of Pediatric Metabolism and Inflammatory Diseases, Chongqing, China

**Keywords:** immunoglobulin A vasculitis, inflammation indicators, multiple systems, neutrophil-to-lymphocyte ratio, platelet-to-lymphocyte ratio

## Abstract

**Background:**

Immunoglobulin A vasculitis (IgAV) is the most common childhood vasculitis, and involvement of multiple systems (gastrointestinal, joint, renal) may affect prognosis. Reliable biomarkers for predicting multi-system involvement are needed.

**Objective:**

To investigate the association between routine inflammation-related laboratory indicators and multi-system involvement in children with IgAV.

**Methods:**

A retrospective study included 657 children with IgAV diagnosed according to EULAR/PRINTO/PRES criteria. Patients were categorized into five mutually exclusive subgroups based on clinical phenotypes: skin only (*n* = 75), gastrointestinal (GI, *n* = 96), joint (*n* = 158), kidney (*n* = 31), and multi-system (*n* = 297) involvement. Laboratory parameters including white blood cell (WBC), neutrophils, platelets, neutrophil-to-lymphocyte ratio (NLR), platelet-to-lymphocyte ratio (PLR), albumin, and D-dimer were compared across groups. Generalized additive models (GAM) and ROC curve analysis were used to determine optimal cut-off values for age and disease duration.

**Results:**

Multi-system involvement was the most common phenotype (45.2%). Compared with other subgroups, multi-system involvement patients showed significantly higher WBC (*P* = 0.032), neutrophils (*P* = 0.001), platelets (*P* = 0.002), NLR (*P* = 0.001), PLR (*P* = 0.002), and D-dimer, while albumin was lowest (*P* < 0.001). Pairwise comparisons revealed that NLR and PLR elevations were most pronounced between multi-system and skin-only groups. GAM analysis identified 10 years as the optimal cut-off for onset age; children >10 years had significantly higher renal involvement rates (25.49% vs. 11.39%, *P* < 0.001) and higher NLR (*P* = 0.019) than those ≤10 years. Multivariate logistic regression identified female sex (OR = 1.650, *P* = 0.038), onset age >10 years (OR = 2.160, *P* = 0.036), and longer disease duration (OR = 1.059, *P* = 0.037) as independent risk factors for renal involvement.

**Conclusion:**

Elevated inflammatory markers including WBC, neutrophils, platelets, NLR, PLR, and D-dimer, as well as decreased albumin, are associated with multi-system involvement in children with IgAV, with the most pronounced differences observed between multi-system and skin-only groups. Onset age >10 years is associated with increased renal involvement risk. These routine laboratory indicators, interpreted in conjunction with age and disease duration, may aid in early risk stratification.

## 1 Introduction

Immunoglobulin A vasculitis (IgAV), also known as Henoch-Schönlein purpura (HSP), is the most frequent vasculitis in children, affecting primarily small blood vessels and resulting from immune complex deposition (1). It often manifests as affecting the skin, joints, kidneys, and digestive system. Among them, involvement of systems such as the digestive system, joints, and kidneys can seriously affect the prognosis of pediatric patients (2). Therefore, exploring predictable biomarkers for multi system involvement is crucial.

Although the exact etiology and potential mechanisms of IgAV are not fully understood, it is essentially an inflammatory response occurring in small blood vessels and capillaries. Inflammation can damage tissue cells, increase the fragility and permeability of capillaries, and cause endothelial dysfunction. Previous studies suggested that a causal relationship between circulating inflammatory factors such as CRP, PCT, and interleukin (IL)-8 and IgAV (3). IL-6, IL-1β, monocyte to high-density lipoprotein cholesterol ratio (MHR) and neutrophil to lymphocyte ratio (NLR) have also been found to be elevated in patients with IgAV (4). However, the association between inflammatory indicators and children with IgAV involving multiple systems is not yet clear (5, 6).

However, the ability of inflammation indicators in blood to predict multi systems involvement in children with IgAV is unclear. In our study, we would like to reveal the relationship between routine hematologic and biochemical inflammatory indicators with the occurrence of multi systems damages in IgAV patients, and further find out its importance in the pathogenesis procedures of IgAV.

## 2 Methods

### 2.1 Participants

We included 657 children under the age of 18 who visited Children’s Hospital of Chongqing Medical University for IgAV between January 2017 and June 2026.

The diagnosis of IgAV in children was based on the Ankara 2008 EULAR/PRINTO/PRES classification criteria (7). Multisystem involvement was defined as cutaneous purpura with concurrent involvement of ≥2 extracutaneous organ systems, whereas the gastrointestinal, joint, and kidney groups were defined as isolated involvement of the corresponding named extracutaneous system. Patients were excluded when the patients had severe systemic diseases (such as uncontrolled heart failure, liver failure, active tuberculosis, HIV/AIDS, malignant tumors, etc.), known severe immunodeficiency diseases, and has been treated with systemic corticosteroids or immunosuppressants for a long time (>1 month) for any reason (within 3 months before enrollment in this study), they are excluded from the study. The inclusion and exclusion criteria can be found in the supplementary (Supplementary Table S1).

We divided all selected patients into five subgroups based on clinical presentation: skin only group, joint involvement group, kidney involvement group, gastrointestinal involvement group, and multi system involvement group (Supplementary Table S2). The research protocol complies with the Declaration of Helsinki (8).

### 2.2 Data collection

All data were collected from electronic medical records, including patient demographics and disease characteristics, medical history, laboratory test results, treatment, and disease progression during hospitalization.

Platelet-to-lymphocyte ratio values were determined by dividing the platelet count by the lymphocyte count, and NLR values were computed by dividing the neutrophils count by the lymphocyte count (3).

### 2.3 Statistical analysis

The normality of continuous variables was assessed using quantile-quantile (Q-Q) plots. Normally distributed continuous variables were presented as mean ± standard deviation (SD), and inter-group comparisons were performed using independent sample t-tests or one-way analysis of variance (ANOVA), with pairwise comparisons conducted using the Student-Newman-Keuls (SNK) q-test. Non-normally distributed continuous variables were presented as median (interquartile range, IQR), and inter-group comparisons were performed using the Mann–Whitney U test or Kruskal–Wallis test, with pairwise comparisons adjusted using the Dunn–Bonferroni method. Categorical variables were presented as frequencies and percentages (%), and inter-group comparisons were performed using the *χ*^2^ test. The optimal cut-off value for onset of time was determined using receiver operating characteristic (ROC) curve analysis, and the optimal cut-off value for onset age was determined using generalized additive models (GAM) (Supplementary Figure S1). Variables with *P* < 0.10 in univariate analysis, along with potential confounders (sex, onset age, and onset of time), were entered into multivariate logistic regression models to identify factors associated with renal involvement and time to first remission of skin purpura (Supplementary Tables S3, S4). All analyses were performed using SAS 9.4. A two-sided *P* < 0.05 was considered statistically significant.

## 3 Results

The baseline demographic, clinical characteristics, disease features, and laboratory parameters of the study population are detailed in Table 1. A total of 657 children with IgAV were enrolled. The distribution of clinical phenotypes was as follows: 11.4% (*n* = 75) showed skin-only involvement, 14.6% (*n* = 96) showed GI involvement, 24.0% (*n* = 158) showed joint involvement, 4.7% (*n* = 31) showed renal involvement, and 45.2% (*n* = 297) showed multi-system involvement, with the multi-system phenotype being the most common. In the overall cohort, there were 365 males (55.6%) and 292 females (44.4%), the mean age at onset was 6.72 ± 2.55 years, and the median disease duration (onset of time) was 5 days.

**Table 1:** Baseline characteristics and laboratory parameters of pediatric IgAV patients across clinical phenotypes.

Variables	Skin involvement (*n* = 75)	GI involvement (*n* = 96)	Joint involvement (*n* = 158)	Kidney involvement (*n* = 31)	Multi-systemic involvements (*n* = 297)	F/*χ*^2^/H	*P*
General information							
Male	40 (53.33)	59 (61.46)	91 (57.59)	15 (48.39)	160 (53.87)	2.757	0.599
Female	35 (46.67)	37 (38.54)	67 (42.41)	16 (51.61)	137 (46.13)		
Onset age (year)	7.19 ± 2.83	6.51 ± 2.70^d^	6.37 ± 2.22^d^	7.97 ± 3.29^bce^	6.74 ± 2.50^d^	3.411	0.009
Onset of time (day)	5.00 (3.00,6.00)	5.00 (3.00,7.00)	5.00 (3.00,6.00)^e^	4.00 (2.00,8.00)	5.00 (4.00,7.00)^c^	14.127	0.007
Blood routine							
WBC	7.84 (6.70,10.40)	8.63 (7.05,11.08)	8.74 (7.11,10.48)	7.49 (5.90,9.86)	9.21 (7.25,11.16)	10.568	0.032
PLT	325.56 ± 90.12^d^	348.90 ± 100.19^d^	347.94 ± 87.63^d^	295.90 ± 75.36^abce^	356.87 ± 91.81^d^	4.351	0.002
RBC	4.52 (4.30,4.80)	4.69 (4.36,5.01)^ce^	4.50 (4.31,4.82)^b^	4.61 (4.19,4.88)	4.53 (4.25,4.80)^b^	12.803	0.012
Hemoglobin	122.37 ± 17.24	127.85 ± 15.88	123.23 ± 10.38	126.52 ± 13.11	123.90 ± 11.65	2.810	0.025
Monocyte	0.30 (0.22,0.40)	0.33 (0.22,0.51)	0.35 (0.26,0.44)^d^	0.26 (0.16,0.41)^c^	0.33 (0.24,0.47)	10.344	0.035
Lymphocyte	2.53 (2.10,3.12)	2.45 (1.99,3.29)	2.50 (1.96,3.14)	2.39 (1.89,3.11)	2.28 (1.83,3.00)	7.586	0.108
Neutrophils	4.70 (3.68,6.60)^e^	5.39 (3.38,7.95)	5.42 (4.02,7.40)	4.22 (3.34,6.70)	6.02 (4.25,7.97)^a^	18.287	0.001
PLR	130.77 (98.02,162.75)^e^	132.37 (107.68,184.15)	136.71 (108.98,165.59)	127.55 (96.69,159.16)	152.56 (115.11,191.99)^a^	16.467	0.002
NLR	1.93 (1.31,2.67)^e^	2.07 (1.24,3.71)	2.14 (1.40,3.27)	1.76 (1.23,2.80)	2.56 (1.59,4.02)^a^	19.571	0.001
Liver and kidney function							
Total Protein	71.92 ± 6.64^be^	68.43 ± 7.44^ad^	70.25 ± 5.53	71.23 ± 5.34^be^	68.72 ± 6.31^ad^	5.730	<0.001
Albumin	44.19 ± 3.58^bce^	42.52 ± 4.74^a^	42.35 ± 3.58^a^	43.50 ± 3.25^e^	41.76 ± 4.39^ad^	5.870	<0.001
ALT	13.00 (9.80,15.00)	11.00 (8.30,17.00)	12.00 (9.30,16.00)	13.20 (10.10,15.50)	11.10 (9.00,15.80)	4.917	0.296
AST	25.71 ± 7.69	24.06 ± 6.96	25.44 ± 6.10	23.12 ± 5.13	24.11 ± 5.42	2.481	0.043
Blood Urea Nitrogen	3.90 (3.30,4.70)	4.02 (3.40,4.96)	4.00 (3.40,4.70)	4.00 (3.20,4.90)	4.00 (3.40,4.70)	1.487	0.829
Creatinine	36.00 (32.00,42.00)	36.00 (30.50,40.00)	35.00 (31.00,41.00)	40.00 (32.00,48.00)	34.20 (30.40,40.90)	7.409	0.116
Uric acid	273.16 ± 73.60^d^	305.37 ± 98.56	270.60 ± 67.81^d^	310.48 ± 84.02^ace^	275.21 ± 97.41^d^	3.696	0.006

Post-hoc pairwise comparisons: a vs. Skin involvement, *P* < 0.05; b vs. GI involvement, *P* < 0.05; c vs. Joint involvement, *P* < 0.05; d vs. Kidney involvement, *P* < 0.05; e vs. Multi-systemic involvements, *P* < 0.05.

Onset of time: time interval from symptom onset to hospital presentation (days). NLR: neutrophil-to-lymphocyte ratio; PLR: platelet-to-lymphocyte ratio.

The renal subgroup is small (*n* = 31); consequently, the lowest values observed in this group (e.g., platelets, D-dimer) should be interpreted with caution due to limited statistical power.

Table 1 revealed that onset age was highest in patients with renal involvement (7.97 ± 3.29 years), significantly higher than that in patients with GI involvement (6.51 ± 2.70 years), joint involvement (6.37 ± 2.22 years), and multi-system involvement (6.74 ± 2.50 years) (F = 3.411, *P* = 0.009). Onset of time also differed significantly across subgroups (H = 14.127, *P* = 0.007), with the longest median duration observed in the multi-system group (5 days).

We further compared inflammation-related laboratory indicators across the five clinical phenotypes (Table 2). Patients with multi-system involvement had significantly higher WBC levels compared with skin-only and renal groups (H = 10.568, *P* = 0.032). Platelet counts differed significantly across groups (F = 4.351, *P* = 0.002), with multi-system patients showing the highest levels (356.87 ± 91.81  ×  10^9^ /L) and renal patients the lowest (295.90 ± 75.93 × 10^9^ /L). Neutrophil counts were significantly elevated in the multi-system group (6.02 × 10^9^ /L) compared with the skin-only group (4.70 × 10^9^ /L) (H = 18.287, *P* = 0.001).

**Table 2:** Multivariate logistic regression analysis of factors associated with time to first remission of skin purpura >1 day.

Variables	*β*	Standard error	Wald χ^2^	*P*	OR(95%CI)
Male					1.0(reference)
Female	−0.224	0.202	1.223	0.269	0.800 (0.538,1.188)
Onset age (year)	−0.031	0.042	0.530	0.467	0.970 (0.893,1.053)
*Onset of time*					
<7 days					1.0(reference)
≥7 days	0.481	0.214	5.052	0.025	1.618 (1.064,2.462)
Total Protein	−0.033	0.017	4.021	0.045	0.967 (0.937,0.999)
Blood Urea Nitrogen	0.043	0.037	1.320	0.251	1.043 (0.970,1.122)
Clinical phenotypes					
Skin involvement					1.0(reference)
GI involvement	−1.997	0.388	26.520	<0.0001	0.136 (0.063,0.290)
Joint involvement	−1.624	0.321	25.537	<0.0001	0.197 (0.105,0.370)
Kidney involvement	−1.670	0.548	9.285	0.002	0.188 (0.064,0.551)
Multi-systemic involvements	−1.557	0.288	29.272	<0.0001	0.211 (0.120,0.370)

NLR differed significantly across the five groups (H = 19.571, *P* = 0.001). Pairwise comparisons revealed that NLR in the multi-system group (median 2.56) was significantly higher than in the skin-only group (median 1.93) (*P* < 0.05), but not significantly different from the GI (2.07), joint (2.14), or kidney (1.76) groups. Similarly, PLR showed significant intergroup differences (H = 16.467, *P* = 0.002), with the multi-system group (median 152.56) significantly higher than the skin-only group (median 130.77) (*P* < 0.05).

Regarding albumin, patients with skin-only involvement exhibited the highest level (44.19 ± 3.58 g/L), significantly higher than the GI (42.52 ± 4.74 g/L), joint (42.35 ± 3.58 g/L), and multi-system (41.76 ± 4.39 g/L) groups (F = 5.870, *P* < 0.001). D-dimer levels also differed significantly across phenotypes (H = 18.287, *P* = 0.001).

Multivariate logistic regression (Table 2) revealed that onset of time ≥7 days (OR = 1.618, 95% CI 1.064–2.462, *P* = 0.025) and total protein level (OR = 0.967, 95% CI 0.937–0.999, *P* = 0.045) were independently associated with prolonged purpura remission (>1 day). Compared with the skin-only group, all other phenotypes had significantly shorter remission times: GI (OR = 0.136, *P* < 0.001), joint (OR = 0.197, *P* < 0.001), kidney (OR = 0.188, *P* = 0.002), and multi-system (OR = 0.211, *P* < 0.001).

Generalized additive models revealed a nonlinear relationship between onset age and renal involvement, with a sharp increase in renal involvement prevalence beginning at approximately 10 years of age (Figure 1). Using 10 years as the cut-off, children >10 years (*n* = 51) had significantly higher renal involvement rates (25.49% vs. 11.39%, *χ*^2^ = 8.567, *P* = 0.003) and higher NLR (3.16 vs. 2.21, *P* = 0.019) compared with those ≤10 years (*n* = 660) (Table 3). Multivariate logistic regression (Table 4) identified female sex (OR = 1.650, 95% CI 1.029–2.646, *P* = 0.038), onset age >10 years (OR = 2.160, 95% CI 1.051–4.439, *P* = 0.036), and longer onset of time (per day increase) (OR = 1.059, 95% CI 1.004–1.117, *P* = 0.037) as independent risk factors for renal involvement.

**Figure 1:**
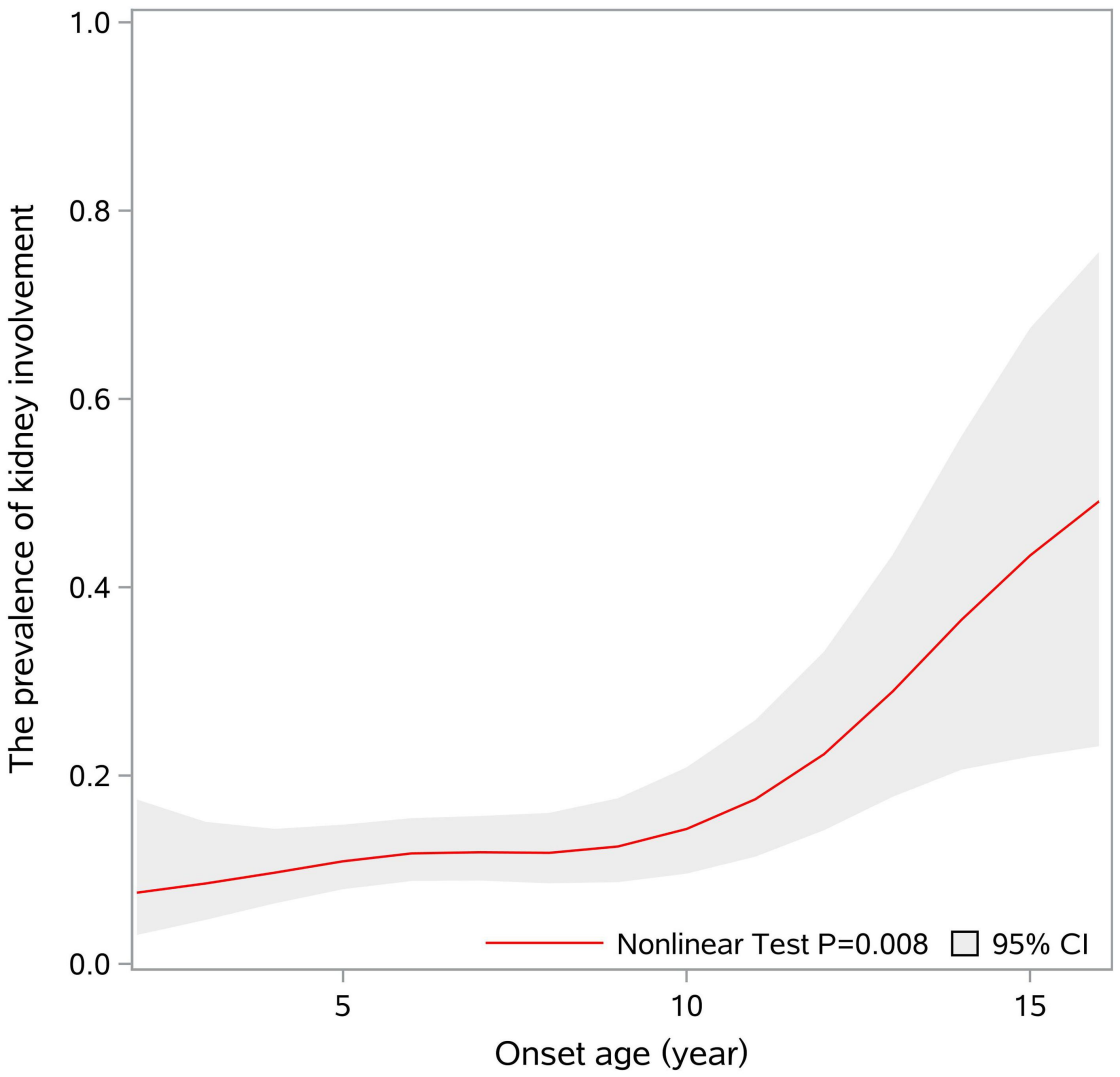
Generalized additive model (GAM) curve of onset age and renal involvement. *X*-axis: onset age (years); *Y*-axis: renal involvement rate (%). The curve shows a sharp increase in renal involvement prevalence beginning at approximately 10 years of age, which was identified as the optimal cut-off. Shaded area represents 95% confidence interval.

**Table 3:** Comparison of baseline characteristics and laboratory indicators of onset age.

Variables	Onset age (year)		t/Z/*χ*^2^	*P*
	≤10 years(*n* = 606)	>10 years(*n* = 51)		
General information				
Male	335 (55.28)	30 (58.82)	0.239	0.625
Female	271 (44.72)	21 (41.18)		
Onset of time (day)	5.00 (3.00,7.00)	6.00 (4.00,9.00)	2.360	0.018
Blood routine				
WBC (×10^9^ /L)	8.86 (7.16,10.89)	8.13 (5.91,11.04)	−1.470	0.141
PLT (×10^9^ /L)	350.74 ± 92.22	303.86 ± 80.19	3.520	<0.001
RBC	4.52 (4.29,4.80)	4.78 (4.57,5.08)	4.261	<0.001
Hemoglobin	123.26 ± 12.40	136.27 ± 13.47	−7.150	<0.001
Monocyte	0.33 (0.24,0.45)	0.28 (0.20,0.40)	−1.918	0.055
Lymphocyte	2.46 (1.93,3.14)	2.08 (1.68,2.36)	−4.316	<0.001
Neutrophils	5.54 (4.04,7.58)	6.10 (3.66,8.26)	0.186	0.853
PLR	138.47 (109.84,178.57)	146.81 (108.26,193.49)	1.163	0.245
NLR	2.21 (1.38,3.58)	3.16 (1.86,4.39)	2.336	0.019
Liver and kidney function				
Total Protein	69.16 ± 6.24	73.89 ± 6.67	−5.171	<0.001
Albumin	42.19 ± 4.18	44.56 ± 3.77	−3.921	<0.001
ALT	11.80 (9.00,15.80)	13.00 (8.10,17.20)	0.276	0.782
AST	24.84 ± 5.80	21.24 ± 8.59	2.932	0.005
Blood Urea Nitrogen	4.00 (3.32,4.71)	4.20 (3.50,5.00)	1.115	0.265
Creatinine	34.35 (30.40,40.00)	51.00 (42.60,58.00)	8.645	<0.001
Uric acid	276.88 ± 87.66	316.28 ± 95.77	−3.060	0.002
Kidney involvement				
No	537 (88.61)	38 (74.51)	8.567	<0.001
Yes	69 (11.39)	13 (25.49)		
Clinical phenotypes				
Skin involvement	68 (11.22)	7 (13.73)	8.406	0.078
GI involvement	88 (14.52)	8 (15.69)		
Joint involvement	153 (25.25)	5 (9.80)		
Kidney involvement	26 (4.29)	5 (9.80)		
Multi-systemic involvements	271(44.72)	26(50.98)		

Data are presented as mean ± SD, median (IQR), or *n* (%). NLR: neutrophil-to-lymphocyte ratio; PLR: platelet-to-lymphocyte ratio.

**Table 4:** Multivariate logistic regression analysis of renal involvement.

Variables	β	Standard error	Wald χ^2^	*P*	OR(95%CI)
Male					1.0(reference)
Female	0.501	0.241	4.316	0.038	1.650 (1.029,2.646)
Onset age (year)					
≤10 years					1.0(reference)
>10 years	0.770	0.367	4.392	0.036	2.160 (1.051,4.439)
Onset of time (day)	0.057	0.027	4.369	0.037	1.059 (1.004,1.117)
AST	−0.012	0.021	0.326	0.568	0.988 (0.949,1.029)
Creatinine	0.008	0.006	1.668	0.196	1.008 (0.996,1.021)

OR, odds ratio; CI, confidence interval. Variables with *P* < 0.10 in univariate analysis were entered into the multivariate logistic regression model. The reference group for onset age was ≤10 years.

## 4 Discussion

IgAV is the most common systemic vasculitis in children, typically presenting as palpable purpura that may involve multiple organ systems, including the gastrointestinal tract, joints, and kidneys (7, 9). Although most pediatric cases are self-limited, mixed IgAV represents a type with increased inflammatory burden and greater risk of complications (10, 11). Identifying readily available laboratory markers reflecting this extensive inflammation is crucial for early risk stratification and appropriate management. In this study, we systematically compared the baseline characteristics and a set of inflammatory markers across different clinical phenotypes of pediatric IgAV, with a particular focus on multisystem involvement (12).

Our data indicate that the mixed phenotype is the most common, accounting for 45.2% of the cohort. Children with mixed IgA vasculitis (IgAV) exhibit pronounced inflammatory characteristics. Their white blood cell count, absolute neutrophil count, and platelet count are significantly higher than those in patients with isolated cutaneous or renal involvement. Furthermore, compared to the abdominal phenotype, the mixed phenotype shows significantly elevated composite inflammation indices, namely neutrophil-to-lymphocyte ratio (NLR) and platelet-to-lymphocyte ratio (PLR). In late-onset children, these ratios are also significantly elevated. Simultaneously, children with the mixed phenotype have the lowest serum albumin levels, and those in the mixed and renal groups exhibit the highest D-dimer levels. In summary, these findings suggest that when multiple organ systems are simultaneously affected in pediatric IgAV, systemic inflammation, coagulation activation, and protein loss are particularly pronounced (13, 14, 17, 18).

Neutrophils and platelets are considered crucial effector cells in small vessel vasculitis. Activated neutrophils release reactive oxygen species, proteolytic enzymes, and neutrophil extracellular traps, directly damaging endothelial cells, while platelets contribute to microvascular thrombosis and amplify the inflammatory cascade by secreting pro-inflammatory mediators (15). NLR and PLR integrate information from both innate and adaptive immune compartments and have been proposed as practical indicators of systemic inflammation and endothelial dysfunction (1, 16). In our cohort, NLR and PLR were significantly elevated in the multi-system group compared with the skin-only group, but not significantly different from the GI, joint, or kidney groups after Bonferroni correction. This pattern suggests that the elevation of NLR and PLR in multi-system disease is primarily driven by the cumulative inflammatory burden of multiple organ involvement, rather than being specific to any single extracutaneous system. Our findings are consistent with previous reports on adult IgA vasculitis, where elevated NLR was associated with gastrointestinal and renal involvement (19, 20, 21).

Albumin is a negative acute-phase protein and a major antioxidant in the body. In various vasculitic conditions, hypoalbuminemia is associated with severe disease and poor prognosis (15). In this study, albumin level was highest in the skin-only group and lowest in the multi-system group, showing a stepwise decrease. This decrease may be multifactorial: persistent systemic inflammation inhibits hepatic albumin synthesis, concurrent gastrointestinal involvement may lead to protein-losing enteropathy, and renal involvement may result in urinary protein loss. Notably, the albumin level in the multi-system group (41.76 ± 4.39 g/L) was even lower than that in the isolated renal group (43.50 ± 3.25 g/L), suggesting that gastrointestinal protein loss may be an important but underrecognized contributor to hypoalbuminemia in children with extensive disease (13). Therefore, close monitoring of serum albumin may provide early indications of developing multiorgan involvement.

Our age stratification analysis using GAM revealed that onset age had a nonlinear relationship with renal involvement, with a sharp increase in risk beginning at approximately 10 years. Children >10 years of age had significantly higher NLR and significantly higher renal involvement rates compared with younger children. This finding aligns with the widely accepted view that older age at onset is a risk factor for IgAV nephritis (1, 22). The more pronounced inflammatory response in older children may be attributed to maturational changes in the immune system-specifically, the physiological transition from lymphocyte predominance to neutrophil predominance that occurs during childhood-or distinct environmental triggers. Furthermore, multivariate logistic regression identified female sex (OR = 1.650, *P* = 0.038), onset age >10 years (OR = 2.160, *P* = 0.036), and longer disease duration (OR = 1.059 per day increase, *P* = 0.037) as independent risk factors for renal involvement. The finding that female sex and longer pre-admission disease duration were also independently associated with renal involvement warrants further validation in prospective studies.

This study has several limitations. Firstly, the retrospective single-center design may limit the generalizability of our findings. Secondly, the sample size of the renal subgroup (*n* = 31) is relatively small, which limits statistical power for detecting differences involving this phenotype; therefore, the lowest values observed in the renal group (e.g., platelets, D-dimer) should be interpreted with caution. Thirdly, we did not systematically collect treatment data; therefore, we could not evaluate the impact of corticosteroid or immunosuppressive therapy on laboratory parameters or time to purpura resolution. Fourthly, lipid profiles and monocyte-to-high-density lipoprotein cholesterol ratio (MHR) were not measured, precluding analysis of the recently emphasized interaction between inflammation and lipid metabolism in IgAV. Fifthly, although we applied Bonferroni correction for multiple comparisons across the seven primary laboratory parameters, some of the observed associations at marginal significance levels should be interpreted with caution. Sixthly, while the GAM-derived 10-year cut-off provides a data-driven threshold for age stratification, external validation in independent pediatric cohorts is needed.

## 5 Conclusion

In conclusion, this study suggested that patients with multi-system involvement exhibited significant inflammatory states characterized by elevated white blood cell, neutrophil, and platelet counts, elevated NLR and PLR, decreased albumin, and increased D-dimer levels. These routine laboratory indicators, interpreted in conjunction with age and disease duration, may aid in early risk stratification. A prospective multicenter study combining treatment adjustment analysis and dynamic biomarker monitoring is needed to validate these findings and improve risk adaptive management strategies for pediatric IgAV.

## Data Availability

The original contributions presented in the study are included in the article/Supplementary Material, further inquiries can be directed to the corresponding author.
