# Supplementary material for: Association of the inflammation indicators with multiple system involved of immunoglobulin A vasculitis in children
Source: Front Pediatr. 2026 Sep 14;14:1921404. doi: 10.3389/fped.2026.1921404 (PMC13617484; doi:10.3389/fped.2026.1921404)
Supplement: Supplementary file 1 [file Supplementaryfile1.docx]

Supplementary Material

# 1 Supplementary Tables

**Supplement Table 1. Inclusion and exclusion criteria for our study**

| **Inclusion** | **Exclusion** |
| --- | --- |
| IgAV diagnostic criteria  1.Palpable purpura, not thrombocytopenic/petechiae  2.Diffuse abdominal pain  3.Histopathology  Typical LCV with predominant IgA deposits or proliferative glomerulonephritis with predominant IgA deposits  4.Arthritis or arthralgias  5.Renal involvement  Proteinuria: >0.3g/24h or >30 mmol/mg of urine albumin to creatinine ratio on a spot morning sample;  Hematuria, red blood cell casts: >5 red cells per high power field or≥2+ on dipstick or red blood cell casts in the urinary sediment | Skin purpura caused by trauma, idiopathic thrombocytopenic purpura, vasculitis and other factors  Abdominal pain caused by acute abdominal symptoms, chronic gastroenteritis, urticaria, etc  Joint pain caused by gout, rheumatoid / rheumatoid arthritis, ankylosing spondylitis, osteoarthritis, developmental deformity and other factors  Kidney diseases caused by nephrotic syndrome, lupus, hepatitis B,diabetes, nephritis, severe infection, tumor, drugs and other factors |

*1 and one of the following

**Supplement Table 2. Different clinical phenotypes of IgAV**

| **Clinical phenotype** | **Criteria** |
| --- | --- |
| **Skin involvement** | Only skin palpable purpura was present, and evidence of other system involvement was excluded |
| **GI involvement** | Skin palpable purpura with digestive system symptomsabdominal pain, stool occult blood positive, intussus  ception, intestinal obstruction, intestinal perforation, etc.) |
| **Joint  involvement** | Skin palpable purpura with swelling, pain / tenderness or arthritis in and around joints, with limited mov  ement |
| **Kidney involvement** | Skin palpable purpura with kidney injury (hematuria, proteinuria, tubular urine, hypertension, etc.) |
| **Multi-systemic  involvement** | Cutaneous purpura with concurrent involvement of at least two of the following extracutaneous systems: gastrointestinal, joint, and renal. |

**Supplement Table 3. Univariate logistic regression analysis of factors associated with time to first remission of skin purpura >1 day**

| **Variables** | **Day to first remission of skin purpura** | | **t/Z/χ2** | **P** |
| --- | --- | --- | --- | --- |
|  | **≤1 day(n=514)** | **>1 day(n=143)** |  |  |
| ***General Information*** | | | | |
| **Male** | 280(54.47) | 85(59.44) | 1.117 | 0.290 |
| **Female** | 234(45.53) | 58(40.56) |  |  |
| **Onset age (year)** | 6.76±2.54 | 6.61±2.65 | 0.627 | 0.531 |
| **Onset of time (day)** | 5.00(3.00,7.00) | 6.00(3.00,8.00) | 1.802 | 0.071 |
| ***Blood routine*** | | | | |
| **WBC** | 8.79(7.08,10.91) | 8.98(6.98,11.03) | 0.069 | 0.945 |
| **PLT** | 347.99±90.02 | 343.93±99.71 | 0.466 | 0.642 |
| **RBC** | 4.55(4.31,4.84) | 4.48(4.23,4.79) | -1.523 | 0.128 |
| **Hemoglobin** | 124.44±13.42 | 123.65±11.15 | 0.714 | 0.476 |
| **Monocyte** | 0.33(0.24,0.46) | 0.33(0.23,0.43) | -0.046 | 0.963 |
| **Lymphocyte** | 2.40(1.89,3.08) | 2.41(1.93,3.10) | 0.034 | 0.973 |
| **Neutrophils** | 5.55(4.06,7.58) | 5.67(3.86,7.63) | -0.416 | 0.677 |
| **PLR** | 140.75(109.88,180.27) | 136.10(105.48,182.86) | -0.413 | 0.679 |
| **NLR** | 2.24(1.41,3.69) | 2.21(1.38,3.77) | -0.243 | 0.808 |
| ***Liver and kidney function*** | | | | |
| **Total Protein** | 69.77±6.33 | 68.65±6.61 | 1.851 | 0.065 |
| **Albumin** | 42.38±4.24 | 42.37±4.04 | 0.024 | 0.981 |
| **ALT** | 11.90(9.00,16.00) | 12.00(8.90,16.00) | -0.514 | 0.608 |
| **AST** | 24.59±6.09 | 24.43±6.30 | 0.274 | 0.784 |
| **Blood Urea Nitrogen** | 3.90(3.32,4.70) | 4.36(3.40,4.90) | 2.051 | 0.040 |
| **Creatinine** | 35.00(31.00,41.00) | 35.00(31.00,40.90) | 0.273 | 0.785 |
| **Uric acid** | 279.26±87.24 | 282.37±94.78 | -0.370 | 0.712 |
| ***Kidney involvement*** | | | | |
| **No** | 451(87.74) | 124(86.71) | 0.109 | 0.742 |
| **Yes** | 63(12.26) | 19(13.29) |  |  |
| ***clinical phenotypes*** | | | | |
| **Skin involvement** | 38(7.39) | 37(25.87) | 39.416 | <0.001 |
| **GI involvement** | 82(15.95) | 14(9.79) |  |  |
| **Joint involvement** | 131(25.49) | 27(18.88) |  |  |
| **Kidney involvement** | 26(5.06) | 5(3.50) |  |  |
| **Multi-systemic involvements** | 237(46.11) | 60(41.96) |  |  |

**Supplement Table 4. Univariate logistic regression analysis of renal involvement**

| **Variables** | **Kidney involvement** | | **t/Z/χ2** | **P** |
| --- | --- | --- | --- | --- |
|  | **No (n=575)** | **Yes (n=82)** |  |  |
| ***General Information*** | | | | |
| **Male** | 328(57.04) | 37(45.12) | 4.131 | 0.042 |
| **Female** | 247(42.96) | 45(54.88) |  |  |
| **Onset age (year)** | 6.61±2.45 | 7.55±3.15 | -2.587 | 0.011 |
| **Onset of time (day)** | 5.00(3.00,7.00) | 5.00(3.00,7.00) | 0.495 | 0.620 |
| ***Blood routine*** | | | | |
| **WBC** | 8.79(7.05,10.89) | 8.97(6.76,11.07) | -0.187 | 0.852 |
| **PLT** | 349.07±92.77 | 333.32±86.99 | 1.450 | 0.148 |
| **RBC** | 4.54(4.30,4.84) | 4.55(4.28,4.81) | -0.441 | 0.659 |
| **Hemoglobin** | 124.30±12.72 | 124.01±14.58 | 0.190 | 0.849 |
| **Monocyte** | 0.33(0.24,0.45) | 0.31(0.22,0.46) | -0.850 | 0.395 |
| **Lymphocyte** | 2.41(1.91,3.08) | 2.31(1.83,3.21) | -0.659 | 0.510 |
| **Neutrophils** | 5.53(3.99,7.58) | 5.82(4.08,7.54) | 0.443 | 0.658 |
| **PLR** | 137.96(109.73,180.27) | 146.74(107.47,183.73) | 0.372 | 0.710 |
| **NLR** | 2.23(1.38,3.67) | 2.24(1.49,3.99) | 0.651 | 0.515 |
| ***Liver and kidney function*** | | | | |
| **Total Protein** | 69.48±6.40 | 69.84±6.43 | -0.473 | 0.636 |
| **Albumin** | 42.43±4.21 | 41.96±4.03 | 0.948 | 0.343 |
| **ALT** | 11.50(9.00,16.00) | 12.80(10.00,15.50) | 1.148 | 0.251 |
| **AST** | 24.69±6.25 | 23.63±5.12 | 1.693 | 0.093 |
| **Blood Urea Nitrogen** | 4.00(3.40,4.80) | 4.00(3.20,4.60) | -0.974 | 0.330 |
| **Creatinine** | 35.00(31.00,40.90) | 38.00(32.00,44.00) | 1.941 | 0.052 |
| **Uric acid** | 278.68±87.17 | 288.76±100.13 | -0.961 | 0.337 |

**Supplement Table 5. Comparison of baseline characteristics and laboratory indicators of onset age**

| **Variables** | **Onset age (year)** | | **t/Z/χ2** | **P** |
| --- | --- | --- | --- | --- |
|  | **≤5 years(n=116)** | **>5 years(n=541)** |  |  |
| ***General Information*** | | | | |
| **Male** | 70(60.34) | 295(54.53) | 1.309 | 0.253 |
| **Famale** | 46(39.66) | 246(45.47) |  |  |
| **Onset of time (day)** | 5.00(3.00,7.00) | 5.00(3.00,7.00) | -0.712 | 0.476 |
| ***Blood routine*** | | | | |
| **WBC (×109/L)** | 8.77(6.97,11.18) | 8.79(7.09,10.82) | -0.165 | 0.869 |
| **PLT (×109/L)** | 380.09±98.04 | 340.03±89.36 | 4.304 | <0.001 |
| **RBC** | 4.46(4.22,4.73) | 4.57(4.31,4.84) | -2.175 | 0.030 |
| **Hemoglobin** | 119.82±10.28 | 125.22±13.27 | -4.857 | <0.001 |
| **Monocyte** | 0.36(0.24,0.51) | 0.33(0.23,0.44) | 1.519 | 0.129 |
| **Lymphocyte** | 2.95(2.21,3.89) | 2.35(1.85,2.94) | 5.703 | <0.001 |
| **Neutrophils** | 5.01(3.45,6.97) | 5.66(4.08,7.63) | -2.510 | 0.012 |
| **PLR** | 126.45(101.87,162.45) | 143.88(110.47,183.33) | -2.883 | 0.004 |
| **NLR** | 1.59(1.06,2.70) | 2.39(1.53,3.81) | -5.274 | <0.001 |
| ***Liver and kidney function*** | | | | |
| **Total Protein** | 66.64±6.71 | 70.15±6.16 | -5.484 | <0.001 |
| **Albumin** | 41.37±4.87 | 42.59±4.01 | -2.518 | 0.013 |
| **ALT** | 11.85(9.00,15.35) | 11.90(9.00,16.00) | -0.782 | 0.434 |
| **AST** | 26.84±6.03 | 24.07±6.05 | 4.492 | <0.001 |
| **Blood Urea Nitrogen** | 3.70(3.20,4.51) | 4.00(3.40,4.80) | -3.278 | 0.001 |
| **Creatinine** | 29.00(25.75,33.00) | 37.00(32.00,42.00) | -10.657 | <0.001 |
| **Uric acid** | 268.36±90.36 | 282.42±88.43 | -1.548 | 0.122 |
| ***Kidney involvement*** | | | | |
| **No** | 109(93.97) | 466(86.14) | 5.359 | 0.021 |
| **Yes** | 7(6.03) | 75(13.86) |  |  |
| ***clinical phenotypes*** | | | | |
| **Skin involvement** | 13(11.21) | 62(11.46) | 3.219 | 0.522 |
| **GI involvement** | 20(17.24) | 76(14.05) |  |  |
| **Joint involvement** | 32(27.59) | 126(23.29) |  |  |
| **Kidney involvement** | 3(2.59) | 28(5.18) |  |  |
| **Multi-systemic involvements** | 48(41.38) | 249(46.03) |  |  |

**Supplement Figure 1. Receiver operating characteristic (ROC) curve of onset of time for predicting time to first remission of skin purpura >1 day**

Supplement Figure 1.The Youden index identified 7 days as the optimal cut-off. AUC=0.549, 95% CI 0.493–0.605.
